# Integrated computational and experimental approach to identify Nrf2-regulated molecular targets in cerebral ischemia

**DOI:** 10.1007/s43440-025-00792-9

**Published:** 2025-10-15

**Authors:** Anita Lewczuk, Anna Boratyńska-Jasińska, Łukasz Charzewski, Małgorzata Beręsewicz-Haller, Barbara Zabłocka

**Affiliations:** 1 Molecular Biology Unit, Mossakowski Medical Research Institute, Polish Academy of Sciences, Warsaw, Poland; 2 Proacta S.A., Warsaw, Poland

**Keywords:** Ischemia/reperfusion, Hippocampus, CA2-3, DG, Nrf2, Gerbil, Regional vulnerability

## Abstract

**Background:**

The transcription factor nuclear factor erythroid 2-related factor 2 (Nrf2) is a master regulator of the cellular antioxidant response, playing an important role in protecting neurons from ischemic injury. The hippocampus exhibits region-specific vulnerability to ischemia, with CA1 neurons being highly susceptible, while CA2-3 and dentate gyrus (DG) neurons demonstrate greater resistance. Our previous work revealed higher basal and post-ischemia/reperfusion (I/R) Nrf2 activity in the resistant CA2-3,DG region compared to CA1. This study aimed to identify potential Nrf2-regulated genes that contribute to this regional neuroprotection in a gerbil model of global cerebral ischemia.

**Methods:**

We used a combined computational and experimental approach. By utilizing the mouse Hipposeq database and Nrf2 target gene lists from the GSEA Molecular Signatures Database, we identified 15 candidate genes with predicted roles in the CA2-3,DG stress response. Quantitative real time-PCR and Western blot analysis were then used to validate expression patterns in the gerbil hippocampus following I/R.

**Results:**

The analysis confirmed distinct expression patterns. Although some genes, including *MPP3*,* RET*, and *SHISA2*, showed higher basal expression in CA2-3,DG, they were unexpectedly downregulated after I/R. In contrast, others, such as *AIFM2*, *BRIP1*, and *CAMK1*, were specifically upregulated in this region. Furthermore, some (*GPC1*) showed delayed upregulation or showed altered protein levels despite unchanged mRNA expression (*FZD7*, *STC2*).

**Conclusions:**

These results emphasize the regional and time-dependent regulation of gene expression in the hippocampus after I/R. The identified up- and downregulated genes represent novel molecular targets whose pharmacological modulation could enhance endogenous neuroprotective pathways, revealing new therapeutic avenues for stroke.

**Supplementary Information:**

The online version contains supplementary material available at 10.1007/s43440-025-00792-9.

## Introduction

Nuclear factor-erythroid 2-related factor 2 (Nrf2) is a crucial transcription factor for a complex cellular defense system against oxidative and electrophilic stress [1]. Under basal conditions, Nrf2 is sequestered in the cytoplasm by its repressor protein, Kelch-like ECH-associated protein 1 (Keap1), which targets Nrf2 for ubiquitination and proteasomal degradation [2]. However, under stress conditions, such as oxidative stress, Nrf2 is released from Keap1, allowing its translocation to the nucleus. Nuclear Nrf2 binds to antioxidant response elements (AREs) in the promoter regions of its target genes, initiating their transcription [3]. The Nrf2 regulon includes hundreds of genes that are involved in various processes: antioxidant and anti-inflammatory responses, heme and iron metabolism, regulation of growth factors and other transcription factors, and metabolic adaptation [4, 5].

The hippocampus, a brain structure essential for learning and memory, is an area where the protective functions of transcription factors can be particularly significant, especially given its striking regional variations in vulnerability to transient cerebral ischemia. The different subregions of the hippocampus, including the Cornu Ammonis areas (CA1, CA2, and CA3) and the dentate gyrus (DG), exhibit distinct morphological and functional characteristics, as well as differential susceptibility to insults such as Alzheimer’s disease, stroke and stress [6, 7]. Transient cerebral ischemia selectively damages CA1 pyramidal neurons, leading to delayed neuronal death, while leaving neurons in CA2, CA3, and DG relatively intact [8, 9]. This selective vulnerability of CA1 manifests itself morphologically within 24 h after a brief ischemic episode, progressing to significant neuronal loss within 4–7 days [10, 11]. Mongolian gerbils have a unique cerebrovascular anatomy, specifically an incomplete circle of Willis, which makes them highly susceptible to experimentally induced cerebral ischemia, especially within the hippocampus [12, 13]. This characteristic makes them an excellent model for investigating stroke mechanisms, neuroprotection, and post-ischemic recovery in this specific brain region, e.g. [14–16]. The mechanisms underlying delayed neuronal death in CA1 have been extensively studied. Recent research, however, has shifted focus to elucidating the factors responsible for the relative resistance of CA2-3 and DG, e.g [17, 18]. The resistance of the CA2-3,DG region likely arises from intrinsic neuroprotective mechanisms, such as enhanced antioxidant defenses, reduced oxidative stress, and increased neurotrophic factors expression, regulated by transcription factors like Nrf2 [19, 20].

Nrf2 plays a crucial role in protecting cells from oxidative stress by inducing the expression of numerous cytoprotective genes [21]. Its activation has emerged as a promising neuroprotective strategy in various neurological disorders, including ischemic stroke, as it alleviates oxidative stress, inflammation, and excitotoxicity [22–24]. Our previous work demonstrated an increase in Nrf2 activity in the gerbil hippocampus after I/R [25], consistent with reports of Nrf2 upregulation in other models of cerebral ischemia [26–28]. Furthermore, our previous work confirmed that I/R induces robust Nrf2 activation in the gerbil hippocampus, demonstrated by Nrf2 nuclear translocation and the significant upregulation of proteins encoded by its canonical target genes, including HO-1, GPx1, and GCLC/M. This activation was more pronounced in the resistant CA2-3,DG region, both basally and after I/R, suggesting a direct link between elevated Nrf2 activity and the endogenous neuroprotection of this region [25]. This is consistent with studies indicating that Nrf2 activation or overexpression provides neuroprotection from ischemic damage in hippocampal neurons [29–31]. Notably, the pharmacological activation of Nrf2 with compounds such as sulforaphane has also been shown to confer neuroprotection in this gerbil model of ischemia [25], reinforcing the therapeutic potential of this pathway.

We employed an integrated computational and experimental approach to investigate this topic further. Using the Hipposeq database [32], a comprehensive transcriptomic resource derived from the mouse hippocampus, combined with curated lists of Nrf2-regulated genes from the GSEA Molecular Signatures Database [33, 34], we identified new putative Nrf2 targets that are likely involved in CA2-3,DG resistance to ischemic injury. These in silico predictions were then validated by quantitative RT-PCR and Western blot analysis, examining the temporal expression profiles of selected genes in the CA1 and CA2-3,DG regions after I/R in Mongolian gerbils. This combined approach aimed to uncover new molecular targets and pathways relevant to ischemic brain injury and to advance our understanding of the complex interaction between Nrf2 signaling and regional responses to ischemia. Furthermore, by utilizing the gerbil model, we sought to explore these mechanisms in a system particularly susceptible to hippocampal ischemia. Our research contributes to ongoing efforts to identify therapeutic targets to improve outcomes after transient ischemic attacks (TIA) and stroke [35, 36].

## Materials and methods

### In silico analysis

Gene expression data were retrieved from the Hipposeq database [32] for each available mouse hippocampal region: dorsal CA1-3 pyramidal cells (PCs), ventral CA1 and CA3 PCs, dorsal and ventral DG granule cells (GCs), and dorsal DG mossy cells (MCs) (Supplementary Figure S1). Using the graphical interface, the data were filtered to generate a list of genes with elevated or diminished expression levels in any region compared to the dorsal CA1 region. Subsequently, the data were further reduced to genes known to be Nrf2-regulated in humans. This selection was carried out employing the NFE2L2.V2 gene set from the GSEA Molecular Signatures Database [37–39].

### Ethical statement and animals

Mongolian gerbils (*Meriones unguiculatus*) were purchased from the animal house of the Mossakowski Medical Research Institute, Polish Academy of Sciences. Animal care was in accordance with ethical guidelines (Directive 86/609/EEC of the European Communities Council). All experimental procedures were approved by the Local Commission for the Ethics of Animal Experimentation no. 2 in Warsaw (WAW2/032/2021). Every effort was made to minimize animal suffering and reduce the number of specimens used.

### Transient brain ischemia in gerbils

Adult male gerbils weighing 60 to 70 g were subjected to transient brain ischemia by bilateral ligation of the common carotid arteries for 5 min under isoflurane anesthesia and controlled normothermic conditions, as previously described [11]. After ischemia, the animals recovered for 24, 48, 72, or 96 h prior to decapitation, and the hippocampal subregions (CA1 and CA2-3,DG) were dissected for the immediate extraction of RNA and proteins. The hippocampi of sham-operated animals served as controls. The animals were randomly selected for the experiments.

### Immunoblotting

For Western blotting, the dissected hippocampal regions were homogenized in an ice-cold cell lysis buffer (Cell Signalling Technology, USA) with 1 mM PMSF (Sigma-Aldrich, Germany) and kept on ice for an additional 5 min for lysis. The samples were sonicated and cleared by centrifugation at 14,000 × g for 10 min at 4 °C. Total protein concentration was determined using the Modified Lowry Protein Assay (Thermo Fisher Scientific, USA). The reduced samples were prepared by boiling at 100 °C for 5 min in Laemmli sample buffer. An equal amount of protein (40 µg) was separated by SDS-PAGE and transferred to a nitrocellulose membrane (Amersham, Cytiva, Germany). After transfer, the membranes were cut horizontally into upper and lower sections based on the expected molecular weights of the target proteins. This strategy was employed to allow for efficient probing of multiple proteins from a single gel without the need for stripping the entire membrane. Additionally, it allowed for optimal antibody incubation for each section while conserving antibody solution. Following total protein imaging, membranes were blocked for 1 h at room temperature with 5% non-fat milk in Tris-buffered saline containing 0.05% Tween 20 (TBST). Subsequently, the membranes were incubated for 2 h at RT with the appropriate primary antibodies diluted in TBST. These included: rabbit polyclonal anti-BRIP1 (1:500; Proteintech, Germany; 24436-1-AP), mouse monoclonal anti-CaMK1 (1:500; Santa Cruz Biotechnology, USA; sc-137225), rabbit polyclonal anti-FSP1 (1:500; Proteintech; 20886-1-AP), rabbit polyclonal anti-FZD7 (1:500; Proteintech; 16974-1-AP), rabbit monoclonal anti-ITGB8 (1:500; Cell Signaling Technology; #88300), rabbit polyclonal anti-LDHB (1:1000; Proteintech; 14824-1-AP), rabbit polyclonal anti-3-PGDH (1:500; Proteintech; 14719-1-AP), rabbit polyclonal anti-SHISA2 (1:1000; Sigma-Aldrich; HPA050172), mouse monoclonal anti-STC2 (1:250; Santa Cruz Biotechnology; sc-293388) and rabbit polyclonal anti-TDO (1:500; Proteintech; 15880-1AP). The membranes were then washed with TBST and incubated for 30 min in RT with corresponding peroxidase-conjugated secondary antibodies: anti-mouse (1:8000; Sigma-Aldrich; A9044) or anti-rabbit (1:4000; Sigma-Aldrich; A0545), diluted in 5% fat-free milk in TBST. Bound antibodies were visualized with Amersham ECL Western Blotting Detection Reagent (Amersham, Cytiva) and signals were captured and quantified using the Fusion FX imaging system (Vilber Lourmat, France). The band intensities of the proteins of interest were normalized to the reference protein L-lactate dehydrogenase B chain (LDHB). After initial probing, membranes were stripped using a mild stripping buffer before being blocked again and reprobed for subsequent primary antibodies or the LDHB loading control. A single representative LDHB blot is shown for illustrative purposes, but quantification was performed for each blot individually.

### Total RNA extraction and cDNA synthesis

Fresh hippocampal sections (approx. 10 mg of tissue) were homogenized in the Fenozol solution supplied by the manufacturer (A&A Biotechnology, Poland) and stored at -80 °C. RNA was isolated using the Total RNA Mini Concentrator kit (A&A Biotechnology), following the manufacturer’s instructions. Quality and concentration were measured with a DeNovix DS-11 FX + spectrophotometer (DeNovix Inc., USA). cDNA was synthesized from 2 µg RNA using the High-Capacity RNA-to-cDNA Kit (ThermoFisher Scientific, USA) in 20-µL reactions. The resulting cDNA was stored at -20 °C until it was required for analysis.

### Primer design and quantitative real-time PCR

The primers were designed using Primer3web version 4.1.0 (https://primer3.ut.ee/; accessed on 2 February 2023) [40] based on available DNA sequences of *Meriones unguiculatus*, verified through primer-BLAST (https://www.ncbi.nlm.nih.gov/tools/primer-blast/; accessed on 3 February 2023), and synthesized by DNA Sequencing and Synthesis Facility (IBB PAS, Poland). The presence of a single peak in the melting curve of each amplicon confirmed the specificity of the primers. The primers used for RT-qPCR are listed in Table 1.

**Table 1:** Primer sequences for quantitative real-time PCR (RT-qPCR)

Gene	Accession number	Primer sequences (5'-3')	Amplicon size (bp)
*GAPDH*	XM_021636934	F: AGTATGACTCTACCCACGGC R: ACTCCACAACATACTCGGCA	150
*HMBS*	XM_021659401	F: GAAGAGTGGCCCAGCTACAG R: CACTGAACTCCTGCTGCTCA	108
*AIFM2*	XM_021649400.1	F: GCCTTGCCCTTCTCACATCT R: CTGCTTCACATGTCCTCGT	120
*BRIP1*	XM_021645850.1	F: GGCATCACCACTGCTACTT R: CTGTATTGCCTCCTCTGAACC	105
*CAMK1*	XM_021637624.1	F: AGAGGACAAGAGGACTCAGAAG R: CATCCAGGGCTACAATGTTAGG	137
*CXCL12*	XM_021662804.1	F: TGACTACAGATGCCCATGC R: TCGGGTCAATGCACACTTGT	147
*FZD7*	XM_021654377.1	F: TGGAGGTGAGGAGAGGTTT R: TGCAAGTCCTAAGCCAGAAG	100
*GPC1*	XM_021632062.1	F: GGAGAATGTTATTGGCAGTGTG R: TGGATGACCTTGGCTGTG	93
*HRK*	XM_021642571.1	F: CGGAGTGTAAAGACCCACCC R: ATAGCATTGGGGTGGCTAGC	95
*ITGB8*	XM_021654173.1	F: AGCTTGGAAGAGTGTACGGC R: CCCCTTCCCAGCCACTAAAG	132
*LRP8*	XM_021661159.1	F: TCTTCACCAACCGACACGAG R: TTGGTAGCCACTTCCACGTC	112
*MPP3*	XM_021642772.1	F: GTAGAGTCCAGCCTCCCTCA R: AAGCGAGGCTTCCCACTAAC	109
*PHGDH*	XM_021632368.1	F: GTAAGGAGGAGCTGATCGCC R: CGCTGCGRRGATGACATCAG	92
*RET*	XM_021652718.1	F: GGTCTCTGTGGACGCTTTCA R: TTCCAAACTCGCCTTCTCCC	101
*SHISA2*	XM_021649050.1	F: TCATCACTGTCCTCCCGGAT R: TTGAGGATGGAGGTGGCAAC	138
*STC2*	XM_021635606.1	F: CGCCCTGGACTTCAATGACT R: TGTAGGGGACTCTCAGGCTC	108
*TDO2*	XM_021663266.1	F: CATGGAACTGCTGTGGAAATAAG R: GGAATGGAGATGATTGCTGTTTAG	101

The RT-qPCR assays were performed in a final reaction volume of 20 µl, containing 100 ng of cDNA, 7 µl of Ambion Nuclease-Free Water (Thermo Fisher Scientific), 10 µl of 2X PowerUp SYBR Green Master Mix (Thermo Fisher Scientific, USA) and 400 nM of each primer. Each sample was run in triplicate on the Applied Biosystems 7500 Fast Real-Time PCR System (Thermo Fisher Scientific). No-template controls were added to each run. The cycling parameters were: 2 min at 50 °C (UDG activation), 2 min at 95 °C (polymerase activation), followed by 40 cycles of 95 °C for 3 s for denaturation and 60 °C for 30 s for annealing. The sets of *BRIP1* and *CXCL12* primers had different annealing temperatures: 62 °C and 58.6 °C, respectively. We included two housekeeping genes, *GAPDH* and *HMBS*, previously validated [41], as a normalization factor for the cycle threshold (Ct) value of each gene of interest. The Ct values were determined using SDS 2.3 software (Applied Biosystems, Thermo Fisher Scientific) and for relative quantification, the mean of triplicates was used. The relative expression ratios of the genes of interest were evaluated using the ΔΔCt method [42] and represented as a fold change in the expression of the calibrator, cDNA from the control gerbil’s brain cortex, set as 1.

### Statistical analysis

All values are expressed as a mean ± standard deviation. Statistical analysis was performed using GraphPad Prism 5.03 (GraphPad Software, San Diego, CA, USA). The significance level of α = 0.05 was selected with ∗ *p* < 0.05, ∗∗ *p* < 0.01, ∗∗∗ *p* < 0.001. Statistical analysis was performed using Student’s t-test to compare means between CA1 and CA2-3,DG controls, or with one-way analysis of variance (ANOVA) followed by Dunnett’s multiple comparison test for ischemia/reperfusion (I/R) groups versus control groups.

## Results

### Nrf2-regulated gene selection in hippocampal subregions

Initial computational analysis, based on the Hipposeq database [32] and curated Nrf2 target gene lists, narrowed the dataset to 129 genes potentially regulated by Nrf2 in hippocampal subregions. The relative gene expression was quantified as the ratio of FPKM (Fragments Per Kilobase per Million mapped fragments) values between the dorsal CA1 region and each of the other regions (Supplementary Figure S2). A total of 31 genes with relative expression coefficients greater than 5 were retained for further analysis. Among these, 10 genes that showed a ratio greater than 40 in at least one region were selected (*BRIP1*, *CXCL12*, *SHISA2*, *STC2*, *ITGB8*, *AIFM2*, *RET*, *PHGDH*, *CAMK1*, *FZD7*). Five additional genes were included, whose relative expression levels, despite not reaching the 40 threshold ratio, were still significantly elevated in certain regions of the hippocampus. These genes included *TDO2* and *HRK* in dorsal DG, *MPP3* in dorsal CA2, *GPC1* in dorsal CA3, and *LRP8 in* MC region (see Supplementary Table 1 for a complete list of genes and their corresponding proteins).

**Fig. 1:**
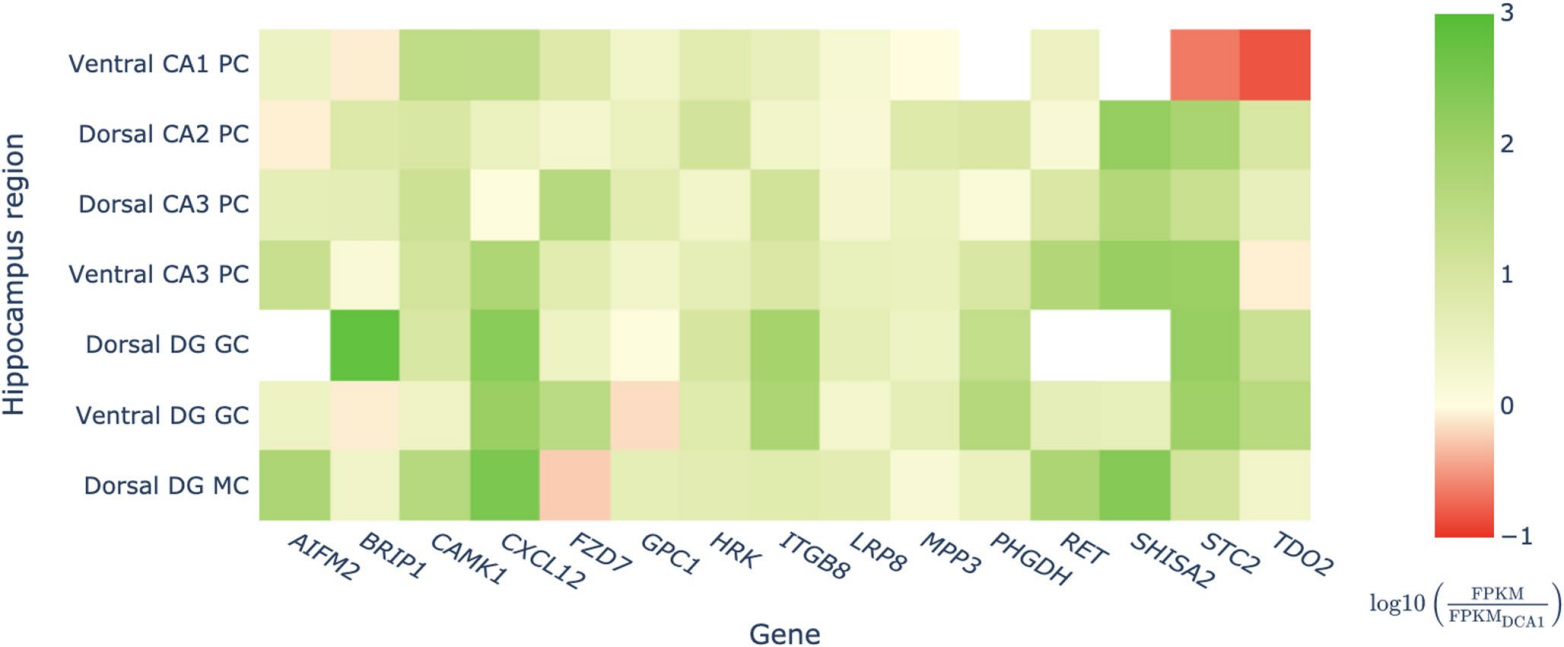
Heatmap representation of relative gene expression in hippocampal subregions - comparison to dorsal CA1. Values represent the base-10 logarithm of the FPKM ratio between the indicated region and the reference region (dorsal CA1). Blank cells indicate missing data in the database, probably due to undetectable or inconsistent expression. Abbreviations: CA1/2/3, Cornu Ammonis areas 1/2/3; DG, dentate gyrus; FPKM, fragments per kilobase per million mapped fragments; GC, granule cells; MC, mossy cells; PC, pyramidal cells

To facilitate visual analysis, the relative expression values were transformed using the base-10 logarithm. A heatmap was then generated using the Plotly graphical library [43] to represent the selected data (Fig. 1).

### Basal gene and protein expression in the gerbil hippocampus

**Fig. 2:**
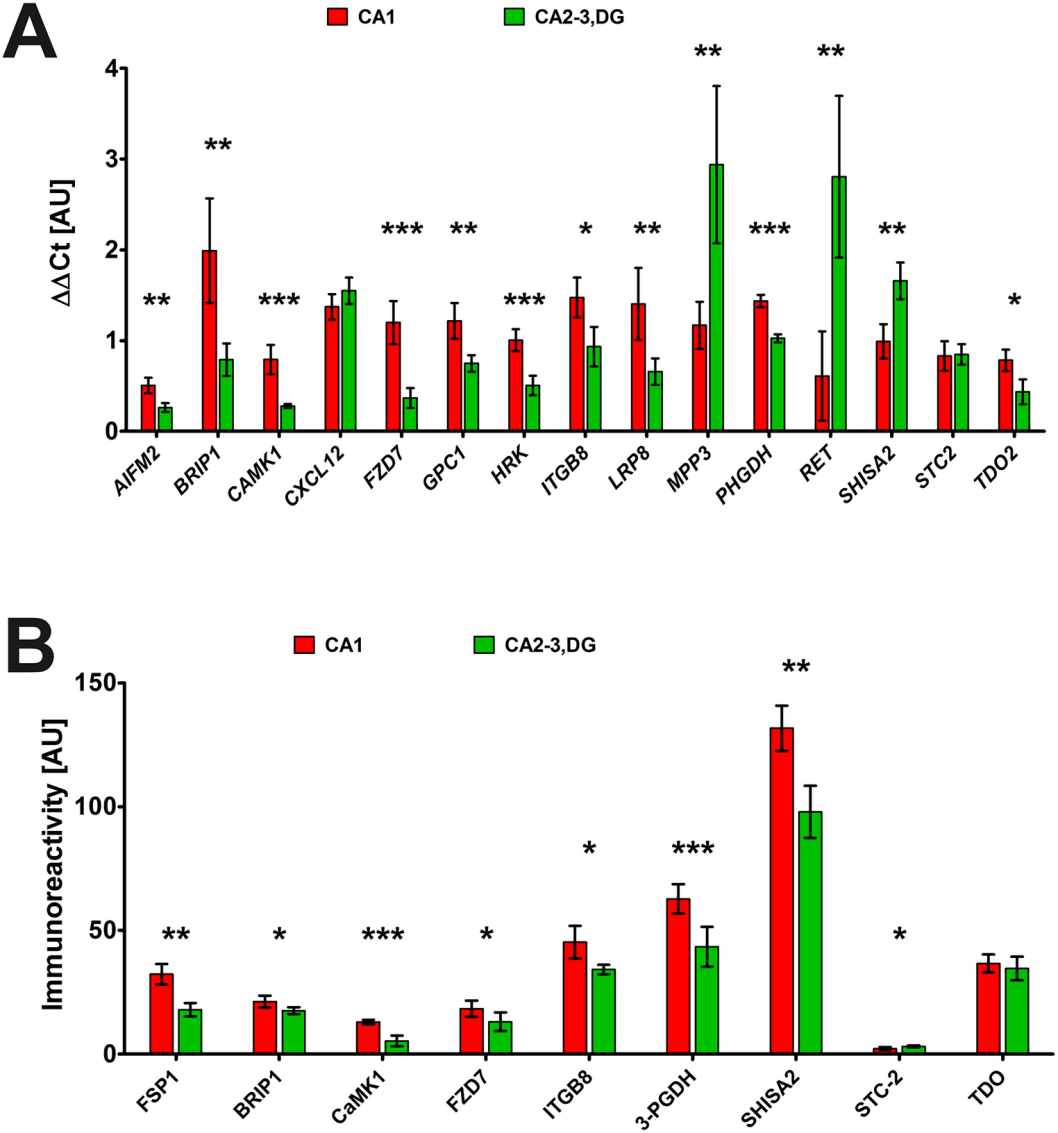
Expression (**A**) and immunoreactivity (**B**) of selected genes and proteins in hippocampal subregions. Expression levels were quantified using the ΔΔCt method, while immunoreactivity levels were normalized to LDHB protein immunoreactivity. Both are expressed in arbitrary units (AU). Data represent mean ± standard deviation (*n* = 3–6). Each gene and protein was analyzed as a separate experiment; statistical significance for each pair was determined using an independent one-sample t-test. *p < 0.05, **p < 0.01, ***p < 0.001. Abbreviations: CA1, Cornu Ammonis area 1; CA2-3,DG, Cornu Ammonis areas 2–3 and dentate gyrus

To validate in silico predictions of elevated expression in the CA2-3,DG region of the hippocampus, we examined the mRNA levels of selected genes in the CA1 and CA2-3,DG regions of control animals using RT-qPCR. Contrary to predictions, only three of the 15 identified genes - *MPP3*, *RET*, *SHISA2* – showed significantly higher expression in CA2-3,DG compared to CA1, which aligns with computational predictions (Fig. 2A). Two genes (*CXCL12* and *STC2*) did not reveal significant interregional differences. The remaining ten genes – *AIFM2*, *BRIP1*, *CAMK1*, *FZD7*, *GPC1*, *HRK*, *ITGB8*, *LRP8*, *PHGDH*, and *TDO2 –* displayed significantly higher expression in CA1, contrasting with the in silico data (full statistical details for all comparisons are provided in Supplementary Table 2). This discrepancy emphasizes the importance of experimental validation of computational predictions in vivo. Additionally, it suggests possible species-specific differences in Nrf2-mediated gene regulation or limitations in computational models. The higher expression of *MPP3*, *RET*, and *SHISA2* mRNA in CA2-3,DG indicates a potential constitutive role for Nrf2 in the regulation of these genes specifically within this hippocampal subregion under basal conditions.

To assess the correlation between mRNA and protein levels, we conducted immunoblotting for a subset of these genes. Due to antibody limitations for Mongolian gerbils, only nine proteins could be analyzed (Fig. 2B). Comparison of mRNA and protein expression revealed a complex, often discordant, relationship. SHISA2, despite significantly elevated mRNA in CA2-3,DG, exhibited higher protein expression in CA1. Similarly, the STC2 protein was elevated in CA2-3,DG, even though there were no significant differences observed in STC2 mRNA. On the contrary, *TDO2* displayed higher mRNA in CA1, but showed equivalent protein levels between regions. For *AIFM2*/FSP1, *BRIP1*, *CAMK1*, *FZD7*, *ITGB8*, and *PHGDH*, both mRNA and protein levels were significantly higher in CA1, suggesting a tighter coupling between transcription and translation for these genes (see Supplementary Table 2 for full statistical details).

Since most of the genes and proteins analyzed showed lower expression in CA2-3,DG than in CA1, we hypothesized that they may not be basally regulated by Nrf2 under normal physiological conditions in this hippocampal subregion. To further explore their potential Nrf2 responsiveness, we examined their expression profiles during an episode of I/R, a condition shown to activate Nrf2 [25, 44].

### Expression of selected genes in hippocampal subregions following ischemia and reperfusion

To assess the involvement of Nrf2 in relative resistance to I/R, we categorized the selected genes into four groups based on their expression profiles in the CA1 and CA2-3,DG regions:

1. Genes with consistent expression patterns: basal expression levels of mRNA were significantly higher in CA2-3,DG compared to CA1, aligning with predictions *in silico.*
2. Genes with delayed upregulation: mRNA basal expression was higher in CA1 but demonstrated significant upregulation specifically in CA2-3,DG following I/R.
3. Genes without significant change in expression in CA2-3,DG after I/R.
4. Genes with inconsistent expression patterns: mRNA expression levels did not match any of the previous categories.

This classification framework enables structured analysis of region-specific transcriptional responses to I/R and facilitates the identification of potential target genes for Nrf2-mediated neuroprotection.

### Temporal expression of MPP3, RET, and SHISA2 in the hippocampus after ischemia/reperfusion

**Fig. 3:**
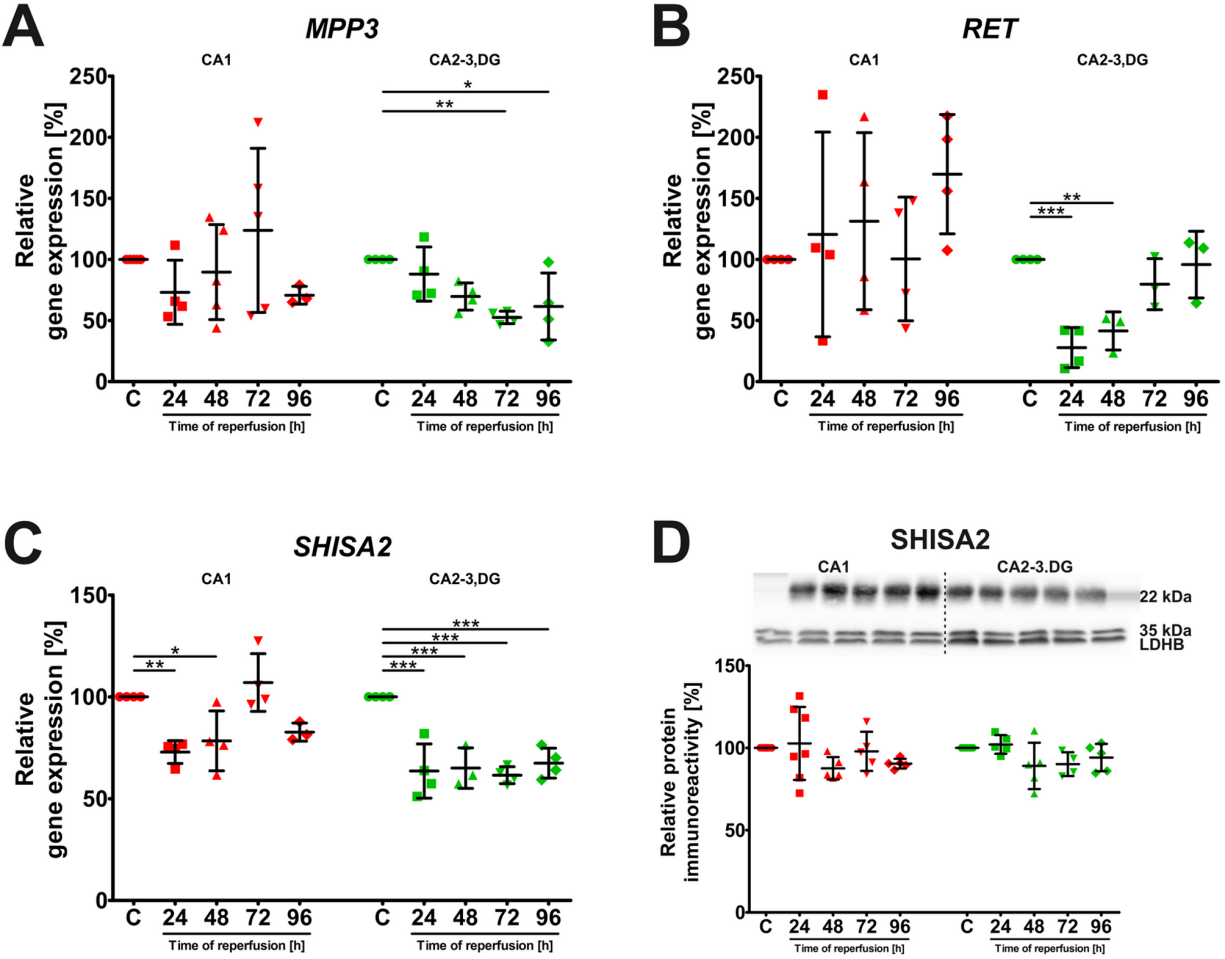
Expression profiles of *MPP3*, *RET*, and *SHISA2* in the hippocampus following I/R. Relative gene expression levels of (**A**) *MPP3*, (**B**) *RET*, and (**C**) *SHISA2* (group of genes exhibiting consistent basal expression patterns) in the CA1 and CA2-3,DG regions of the hippocampus at various time points (24, 48, 72, and 96 h) after 5 min of global cerebral ischemia followed by reperfusion. (**D**) Representative Western blot and densitometric analysis of SHISA2 protein levels in CA1 and CA2-3,DG lysates. SHISA2 protein levels were normalized to LDHB. For each experimental group, proteins were separated on a single gel, transferred to one membrane, and probed sequentially for proteins of interest and the LDHB loading control after stripping. A single, representative LDHB blot is shown for illustrative purposes

Following the categorization of genes based on their basal expression patterns, we examined the temporal changes in expression for the Group 1 genes (*MPP3*, *RET*, and *SHISA2*), which exhibited constitutively higher expression in the CA2-3,DG region compared to CA1 under control conditions. Contrary to expectations of I/R-induced upregulation, none of these genes showed statistically significant increases in expression at any reperfusion time point in CA1 or CA2-3,DG. Instead, a prominent trend of downregulation was observed, particularly in the CA2-3,DG region. The expression of *MPP3* mRNA was significantly decreased in CA2-3,DG at 72 h and 96 h after reperfusion, while remaining relatively stable in CA1 (Fig. 3A). *RET* expression, although not significantly altered in CA1, showed significant downregulation in CA2-3,DG with a marked decrease of approximately 70% at 24 h, followed by a gradual return to baseline levels by 96 h (Fig. 3B). The high variability in *RET* mRNA levels observed in CA1, as indicated by the large standard deviations, contrasted with the consistent response in CA2-3,DG, suggesting a more heterogeneous response to I/R within the CA1 region. *SHISA2* mRNA levels also exhibited significant downregulation in CA2-3,DG at all time points (Fig. 3C), and were significantly reduced in CA1 at 24 and 48 h post-I/R. Despite these robust changes at the mRNA level, SHISA2 protein levels remained relatively stable in both regions of the hippocampus after I/R (Fig. 3D), highlighting the potential influence of post-transcriptional regulatory mechanisms. The observed downregulation of these putatively Nrf2-regulated genes in CA2-3,DG after ischemia is unexpected and warrants further investigation. Full statistical details for the analyses presented in Fig. 3 are provided in Supplementary Table 3.

Data are presented as mean ± standard deviation. Individual data points represent biological replicates (*n* = 4–7). One-way ANOVA followed by Dunnett’s multiple comparison test (all columns vs. control) was performed for each hippocampal region independently. (*p < 0.05, **p < 0.01, ***p < 0.001). Abbreviations: C, control; CA1, Cornu Ammonis area 1; CA2-3,DG, Cornu Ammonis areas 2–3 and dentate gyrus; I/R, ischemia/reperfusion; LDHB, L-lactate dehydrogenase B chain.

### Temporal expression and protein levels of delayed upregulation genes following ischemia and reperfusion

**Fig. 4:**
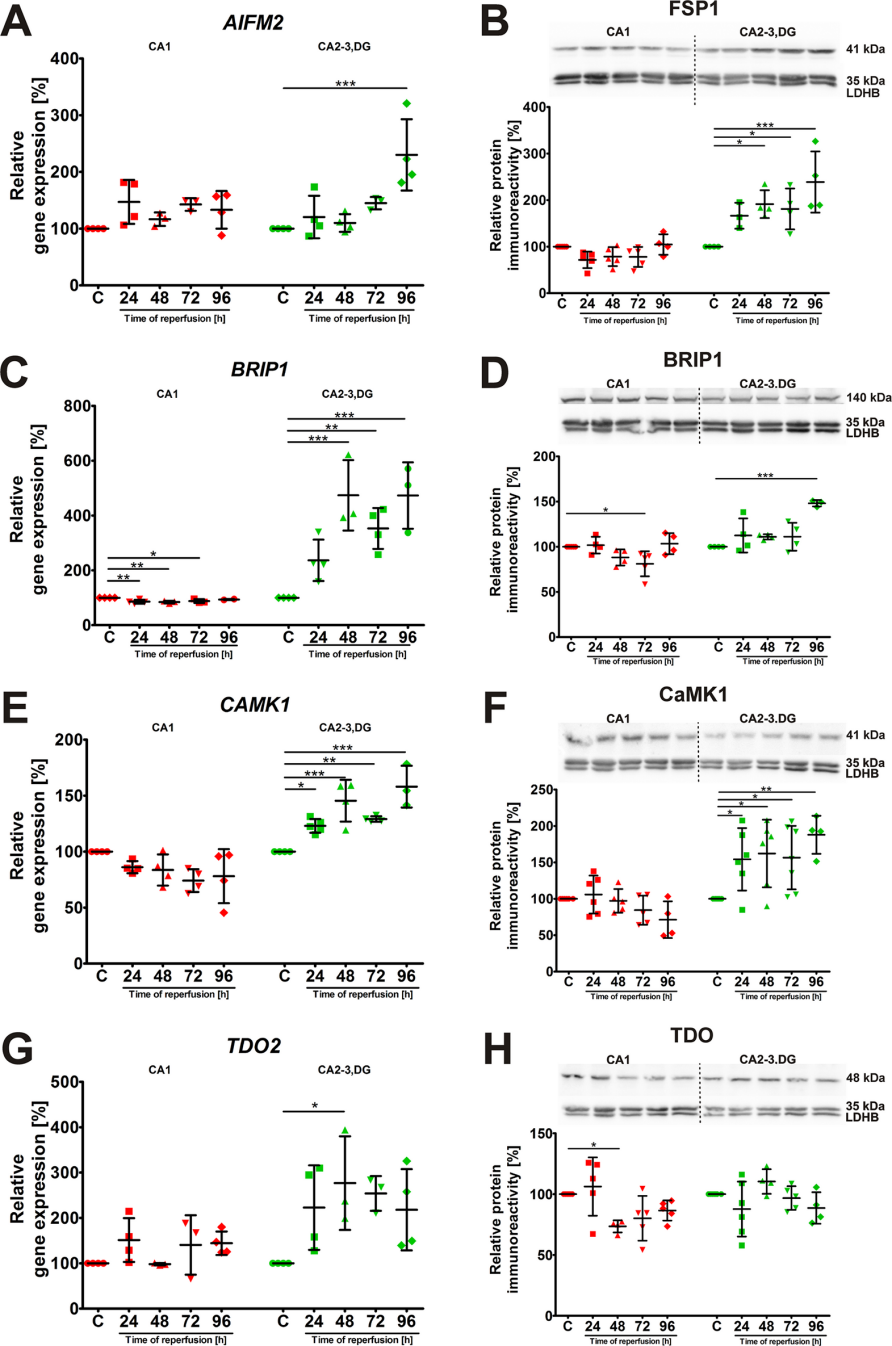
Expression profiles of *AIFM2*, *BRIP1*, *CAMK1*, and *TDO2* in the hippocampus following I/R. Relative gene expression levels of (**A**) *AIFM2*, (**C**) *BRIP1*, (**E**) *CAMK1*, and (**G**) *TDO2* in the CA1 and CA2-3,DG regions of the hippocampus at various time points, after 5 min of global cerebral ischemia followed by reperfusion. These genes showed delayed upregulation in CA2-3,DG after I/R. Representative Western blots and densitometric analyses of (**B**) FSP1 (*AIFM2* protein), (**D**) BRIP1, (**F**) CaMK1, and (**H**) TDO protein levels in CA1 and CA2-3,DG lysates. Protein levels were normalized to LDHB. For each experimental group, proteins were separated on a single gel, transferred to one membrane, and probed sequentially for proteins of interest and the LDHB loading control after stripping. A single, representative LDHB blot is shown for illustrative purposes

Next, the expression profiles of *AIFM2*, *BRIP1*, *CAMK1*, and *TDO2* were examined along with their corresponding proteins. These genes were classified as exhibiting upregulation specifically in the CA2-3,DG region after ischemia. Notably, *AIFM2* mRNA expression significantly increased in CA2-3,DG at 96 h post I/R (Fig. 4A), while remaining unchanged in CA1. Additionally, the upregulation of the FSP1 protein (Fig. 4B) occurred before the increase in mRNA levels, being detectable in CA2-3,DG as early as 48 h and reaching approximately 240% at 96 h. *BRIP1* displayed a sustained and significant increase in mRNA expression in CA2-3,DG starting at 48 h post-ischemia (Fig. 4C). In contrast, in CA1, *BRIP1* mRNA showed a significant decrease. These transcriptional changes were partially reflected at the protein level, with the BRIP1 protein significantly decreasing in CA1 at 72 h and significantly increasing at 96 h in CA2-3,DG (Fig. 4D). Similarly to *BRIP1*, *CAMK1* expression was significantly upregulated in the CA2-3,DG region starting at 24 h and through all analyzed time points of reperfusion (Fig. 4E), while in CA1 a significant decrease was observed at 72 h. CaMK1 protein levels (Fig. 4F) were significantly elevated at every time point in CA2-3,DG and remained relatively stable in CA1, further highlighting the regional differences in response to I/R. Finally, TDO2 mRNA expression in CA2-3,DG showed a trend toward upregulation at all time points post-ischemia, reaching significance at 48 h (Fig. 4G). No relevant changes were observed in the CA1 region. The levels of TDO2 protein remained relatively stable in both regions (Fig. 4H). Full statistical details for the analyses presented in Fig. 4 are provided in Supplementary Table 3.

The delayed upregulation of these genes in the CA2-3,DG region, particularly in the absence of significant changes in the CA1, suggests a specific neuroprotective response to transient ischemic injury. This response may contribute to the enhanced resistance of this region.

Data are presented as mean ± standard deviation. Individual data points represent biological replicates (*n* = 4–7). One-way ANOVA followed by Dunnett’s multiple comparison test (all columns vs. control) was performed for each hippocampal region independently. (*p < 0.05, **p < 0.01, ***p < 0.001). Abbreviations: C, control; CA1, Cornu Ammonis area 1; CA2-3,DG, Cornu Ammonis areas 2–3 and dentate gyrus; I/R, ischemia/reperfusion; LDHB, L-lactate dehydrogenase B chain.

### Temporal expression of FZD7, ITGB8, PHGDH, and STC2 in the hippocampus following ischemia and reperfusion

**Fig. 5:**
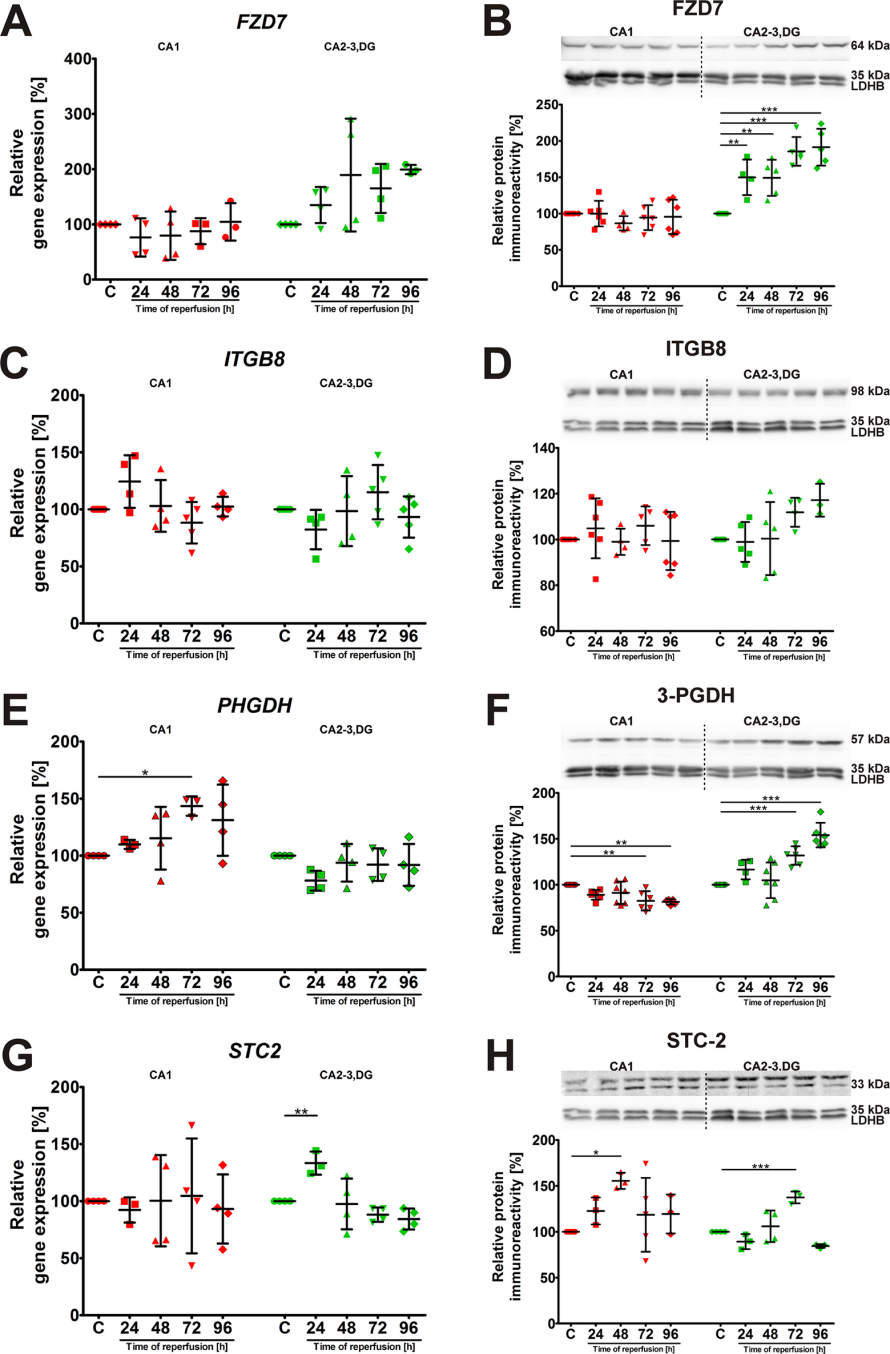
Expression profiles of *FZD7*, *ITGB8*, *PHGDH*, and *STC2* in the hippocampus following I/R. Relative gene expression levels of (**A**) *FZD7*, (**C**) *ITGB8*, (**E**) *PHGDH*, and (**G**) *STC2* in the CA1 and CA2-3,DG regions of the hippocampus at various time points, after 5 min of global cerebral ischemia followed by reperfusion. This group of genes did not show significant changes in expression in CA2-3,DG after I/R. Representative Western blots and densitometric analyses of (**B**) FZD7, (**D**) ITGB8, (**F**) 3-PGDH (PHGDH protein), and (**H**) STC-2 protein levels in CA1 and CA2-3,DG lysates. Protein levels were normalized to LDHB. For each experimental group, proteins were separated on a single gel, transferred to one membrane, and probed sequentially for proteins of interest and the LDHB loading control after stripping. A single, representative LDHB blot is shown for illustrative purposes

The third group of genes we examined was classified as exhibiting no significant change in expression in the CA2-3,DG region following I/R. *FZD7* mRNA expression remained relatively stable in CA1 and CA2-3,DG throughout the reperfusion period, without significant changes (Fig. 5A). However, FZD7 protein levels increased significantly (Fig. 5B) in CA2-3,DG at all times post-ischemia, revealing a disconnection between transcriptional and translational regulation. The expression *of ITGB8* showed a similar pattern, with no significant changes in mRNA levels in both hippocampal regions (Fig. 5C). ITGB8 protein levels also remained relatively constant upon reperfusion (Fig. 5D). *PHGDH* mRNA expression showed a tendency to increase in CA1, reaching significance at 72 h of reperfusion (Fig. 5E), while remaining unchanged in CA2-3,DG. PHGDH protein levels (Fig. 5F), on the contrary, showed a significant decrease in CA1 at 72 and 96 h, while showing a significant upregulation in CA2-3,DG at the same time points. This observation further highlights the possibility of post-transcriptional regulation. The expression of *STC2* did not show statistically significant changes in the CA1 region, but in CA2-3,DG displayed a significant increase at 24 h and returned to baseline later after longer reperfusion time (Fig. 5G). However, STC-2 protein levels showed a significant increase in CA1 at 48 h and in CA2-3,DG at 72 h with a recovery towards baseline levels at later time points (Fig. 5H). Full statistical details for the analyses presented in Fig. 5 are provided in Supplementary Table 3. The lack of significant transcriptional changes in CA2-3,DG for these Group 3 genes, despite the resistance to ischemic injury and the activation of Nrf2 under these conditions, indicates that their regulation is likely independent of the primary Nrf2-mediated neuroprotective response.

Data are presented as mean ± standard deviation. Individual data points represent biological replicates (*n* = 4–7). One-way ANOVA followed by Dunnett’s multiple comparison test (all columns vs. control) was performed for each hippocampal region independently. (*p < 0.05, **p < 0.01, ***p < 0.001). Abbreviations: C, control; CA1, Cornu Ammonis area 1; CA2-3,DG, Cornu Ammonis areas 2–3 and dentate gyrus; I/R, ischemia/reperfusion; LDHB, L-lactate dehydrogenase B chain.

### Temporal expression of genes with inconsistent expression patterns in the hippocampus after ischemia/reperfusion

**Fig. 6:**
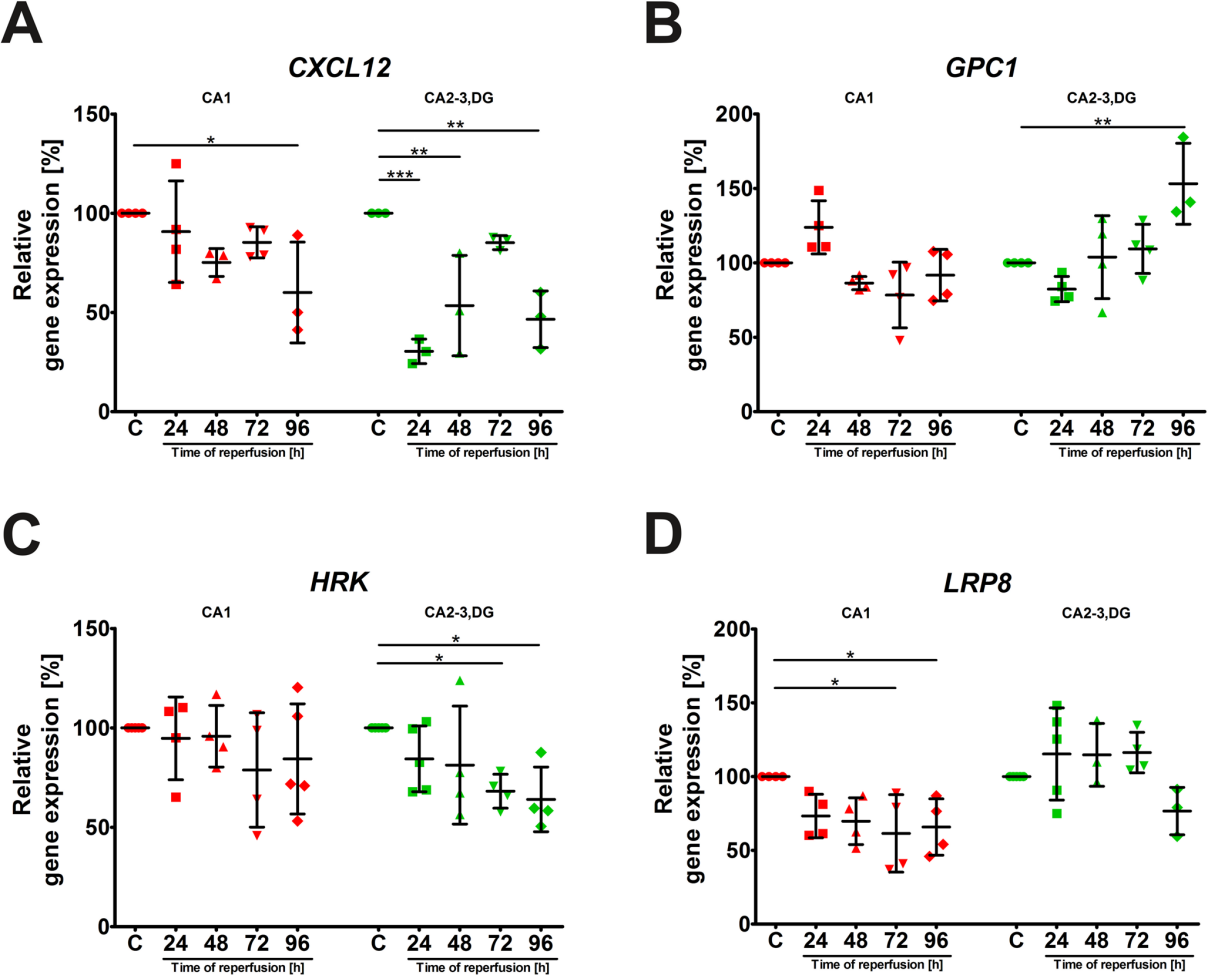
Expression profiles of *CXCL12*, *GPC1*, *HRK*, and *LRP8* in the hippocampus following I/R. Relative mRNA expression levels of (**A**) *CXCL12*, (**B**) *GPC1*, (**C**) *HRK*, and (**D**) *LRP8* in the CA1 and CA2-3,DG regions of the hippocampus at various time points, after 5 min of global cerebral ischemia followed by reperfusion. These genes displayed inconsistent expression patterns that did not fit into the previously defined groups. For each experimental group, proteins were separated on a single gel, transferred to one membrane, and probed sequentially for proteins of interest and the LDHB loading control after stripping. A single, representative LDHB blot is shown for illustrative purposes

Finally, we analyzed the expression profiles of *CXCL12*, *GPC1*, *HRK*, and *LRP8*, classified here as Group 4 due to their varied expression profiles in the CA1 and CA2-3,DG regions. The expression of *CXCL12* mRNA was significantly downregulated in CA1 at 96 h after reperfusion (Fig. 6A). In CA2-3,DG, *CXCL12* was also significantly downregulated at 24 h, its levels fluctuating but generally suppressed throughout the reperfusion period. *GPC1* expression remained relatively stable in CA1 but showed a significant increase in CA2-3,DG at 96 h after I/R (Fig. 6B), suggesting a delayed, region-specific response. *HRK* mRNA levels were significantly downregulated at 72 h and 96 h in the CA2-3,DG region (Fig. 6C), while remaining unchanged in CA1. *LRP8* expression was significantly reduced in CA1 at later times post-ischemia. In the CA2-3,DG region, *LRP8* expression showed a tendency to decline at 96 h after I/R but remained relatively stable (Fig. 6D). Full statistical details for the analyses presented in Fig. 6 are provided in Supplementary Table 3.

Data are presented as mean ± standard deviation. Individual data points represent biological replicates (*n* = 4–7). One-way ANOVA followed by Dunnett’s multiple comparison test (all columns vs. control) was performed for each hippocampal region independently. (*p < 0.05, **p < 0.01, ***p < 0.001). Abbreviations: C, control; CA1, Cornu Ammonis area 1; CA2-3,DG, Cornu Ammonis areas 2–3 and dentate gyrus; I/R, ischemia/reperfusion.

## Discussion

In this study, we investigated the complex transcriptional and post-transcriptional responses in the gerbil hippocampus following I/R. Our principal findings reveal a striking heterogeneity in gene expression across hippocampal subregions. A key observation was the unexpected downregulation of several putative Nrf2 target genes that have high basal expression in the ischemia-resistant CA2-3,DG region. This contrasted with another group of genes that exhibited a delayed and region-specific upregulation specifically in this resilient area. Finally, we frequently observed a discordance between mRNA and protein levels, underscoring the critical role of post-transcriptional regulation in shaping the post-ischemic proteome. These results collectively suggest that the hippocampal response to ischemia is governed by a complex, region-specific interplay of regulatory mechanisms that extends beyond a uniform Nrf2-mediated transcriptional program.

Although the neuroprotective role of Nrf2 is well established [1, 23, 45], the exact downstream genes and pathways that mediate this effect in the hippocampus, particularly after the I/R episode, remain unclear. Several computational studies have attempted to identify Nrf2-regulated genes in the hippocampus using various approaches, such as single-cell RNA sequencing (scRNA-Seq) data analysis, microarray analysis of Nrf2 knockout models, or in vitro cell culture systems exposed to Nrf2 activators [46–49]. However, these studies often lack the context of in vivo ischemic injury and may not adequately reflect the complex cellular responses in the intact brain. In addition, previous in silico analyses have not adequately addressed the differential vulnerability of hippocampal subregions to ischemic damage. Although the CA1 region is highly susceptible to I/R injury, CA2-3,DG exhibits relative resistance [50, 51]. Understanding the molecular mechanisms underlying the relative resistance of CA2-3,DG is of particular interest, as these mechanisms may provide potential therapeutic targets for improving neuroprotection in more vulnerable regions. Our approach used the Hipposeq database [32], a comprehensive transcriptomic resource derived from the mouse hippocampus, and the curated lists of Nrf2-regulated genes [33, 34]. By integrating these datasets, we sought to identify new candidate genes that contribute to resistance of the CA2-3,DG region and subsequently validated their expression patterns in a gerbil model of global cerebral I/R. This was done to gain new insights into the molecular mechanisms underlying Nrf2-mediated neuroprotection in the hippocampus after ischemic injury. Our findings show striking heterogeneity in the temporal and regional expression patterns of Nrf2-regulated genes following I/R, illustrating the complex relationship between transcriptional and post-transcriptional regulatory mechanisms that influence the hippocampal response to ischemic injury.

One of the most intriguing findings of our study was the divergent behavior of the Group 1 genes (*MPP3*, *RET*, and *SHISA2*) in the CA1 and CA2-3,DG regions after I/R. These genes showed higher basal expression in CA2-3,DG, consistent with in silico predictions and potentially implicating Nrf2 in their constitutive regulation. Given our previous findings that I/R induces both increased Nrf2 levels and activity in the CA2-3,DG region [25], we initially anticipated upregulation of these putatively Nrf2-regulated genes in this region. Contrary to expectations, I/R led to a significant downregulation of these genes in CA2-3,DG, particularly at later time points (Fig. 3). *RET* displayed a transient but noticeable decrease at 24–48 h, while both *MPP3* and *SHISA2* were significantly and persistently downregulated in this region. The observed downregulation of Group 1 genes in the CA2-3,DG region suggests interactions among multiple regulatory pathways rather than a straightforward Nrf2-mediated response. This unexpected finding adds complexity to our understanding of neuroprotection in the hippocampus following ischemia and emphasizes the possibility of region-specific regulatory mechanisms.

The reduced expression of Group 1 genes in the more resistant CA2-3,DG region is counterintuitive and deserves further analysis. Several hypotheses may explain these findings. First, MPP3, RET, and SHISA2 may be involved in cellular processes that are beneficial under normal conditions but could become harmful during prolonged ischemic stress. For example, MPP3 encodes a membrane-associated guanylate kinase (MAGUK) protein, a family of proteins known to play a crucial role in establishing and maintaining cell polarity, synapse formation, and neuronal signaling [52]. Specifically, MPP3 has been demonstrated to be required for the maintenance of the apical junctional complex during neuronal migration and cortical development [53]. Its downregulation after I/R could, therefore, reflect a compensatory response aimed at limiting these processes during cellular stress. In the context of the pronounced and prolonged downregulation of RET during reperfusion, selectively in the CA2-3,DG area, it is worth noting that this gene encodes a receptor tyrosine kinase activated by ligands from the GDNF family, which typically promotes neuronal survival and differentiation [54, 55]. Its reduced expression in this context might reflect a region-specific adaptive response. In models of focal ischemia, exogenous administration of GDNF has been established to reduce ischemic brain injury. This protective effect may be mediated, in part, by GDNF’s ability to reduce NMDA receptor-mediated Ca^2+^ influx through an ERK-dependent pathway, thus preventing excitotoxic neuronal death [54]. Therefore, downregulation of RET in CA2-3,DG after I/R could be a mechanism to limit calcium overload and excitotoxicity in this region, potentially reflecting the protective effects of exogenous administration of GDNF. Furthermore, SHISA2 mRNA levels were significantly and persistently downregulated in CA2-3,DG at all time points post-I/R, while remaining relatively unchanged in CA1. This gene encodes a transmembrane protein belonging to the Shisa family, members of which have been shown to interact and regulate AMPA-type glutamate receptors [56, 57]. Given the role of excitotoxicity in ischemic brain injury [58], the downregulation of SHISA2 in the CA2-3,DG region could represent a protective mechanism by dampening glutamatergic signaling and reducing neuronal excitability. Interestingly, despite changes in mRNA levels, SHISA2 protein levels remained unchanged in both CA1 and CA2-3,DG during the study period after ischemia, which might be due to the long half-life or compensatory translational mechanisms.

The sustained downregulation of MPP3, RET, and SHISA2 in CA2-3,DG during reperfusion occurred in a model where we have previously confirmed robust Nrf2 activation [25], indicating that the regulation extends beyond direct Nrf2-mediated transcription. One hypothesis is that this downregulation reflects a region-specific adaptive response, potentially limiting harmful overactivation of certain pathways or reducing the burden on an already stressed system after I/R. For example, it could be related to the initiation of ferroptosis, a form of iron-dependent cell death, which has been shown to occur in CA1 neurons following ischemia, but is less pronounced in the CA3 region [59, 60]. Alternatively, CA2-3,DG neurons may shift metabolic priorities post-ischemia, reallocating resources to pathways more critical for survival and away from functions associated with Group 1 gene products. Moreover, the downregulation could be part of a negative feedback loop, fine-tuning the cellular response to I/R. Initial activation of Nrf2 target genes might trigger neuroprotective processes, such as increased antioxidant defense or the unfolded protein response, which, when reaching a certain threshold, induce feedback inhibition to prevent excessive or prolonged activation, as exemplified by the Nrf2-p97-Nrf2 loop [61] and supported by the broader context of Nrf2 regulation in stroke [62]. Fourthly, it cannot be excluded that while in silico analysis predicted these genes as Nrf2 targets, their regulation may also involve other transcription factors or post-transcriptional mechanisms, particularly in the context of regional stress responses. Lastly, the discordance between SHISA2 mRNA and protein levels highlights the crucial role of post-transcriptional mechanisms, such as regulation of mRNA stability, translational control, or protein degradation [63]. These findings underline the limitations of relying solely on transcriptional profiling, especially during stress when translational and post-translational regulation can significantly alter protein abundance and activity. The observed regional differences also suggest that Nrf2 activity itself could be differentially regulated in CA1 and CA2-3,DG after I/R. Although our previous work showed post-ischemic elevated Nrf2 activity in both regions, though with different dynamics [25], downstream effects appear to diverge, with CA2-3,DG exhibiting a more complex response involving up- and downregulation of putative Nrf2 targets. This could be due to region-specific cofactors or modulators that influence Nrf2 transcriptional activity or the activation of distinct signaling pathways that intersect Nrf2 signaling in CA2-3,DG. Future studies using cell-type-specific analyses would help further address this important issue.

In contrast to Group 1, genes assigned to Group 2 (*AIFM2*,* BRIP1*,* CAMK1*, and *TDO2*) showed a pattern of delayed upregulation specifically in CA2-3,DG following I/R (Fig. 4). This delayed response, particularly evident for *AIFM2* and *BRIP1*, suggests that these genes may be involved in later-stage neuroprotective processes, possibly contributing to the enhanced recovery and repair capacity of CA2-3,DG. The observation that AIFM2 (also known as FSP1) protein levels increased prior to mRNA upregulation (Fig. 4A, B) suggests that AIFM2 may in part be under post-transcriptional control during reperfusion after I/R. Indeed, recent research indicates that AIFM2 acts as a crucial factor in regulating ferroptosis [64]. This regulation depends on the GSH- and NAD(P)H-dependent antioxidant activity of the protein [65]. Therapeutic interventions against ferroptosis have been proposed to be based not only on the inhibition of lipid peroxidation but also on the enhancement of AIFM2 antioxidant activity. Our study also showed that *BRIP1* displayed a sustained and significant increase in mRNA expression in CA2-3, DG, which contrasted with the decrease in CA1. This transcriptional change was partially reflected at the protein level. This observation is consistent with a potential role for Nrf2 in mitigating the effects of I/R injury through the modulation of BRIP1 expression in CA2-3,DG, although we cannot exclude the involvement of other regulatory factors. *BRIP1* encodes a DNA-dependent ATPase and a 5’-3’ DNA helicase required for the maintenance of chromosomal stability and involved in the repair of DNA double-strand breaks by homologous recombination, in a manner dependent on its association with BRCA1 [66, 67]. BRIP1 has also been shown to play an important role in maintaining neuronal cell health and homeostasis by suppressing cellular oxidative stress [68]. Similarly to *BRIP1*, *CAMK1* expression was significantly upregulated in the CA2-3,DG region, while protein levels (Fig. 4F) were significantly elevated at every time point in CA2-3,DG and remained relatively stable in CA1. CaMK1 is an element of the Ca^2+^/CaM-dependent protein kinase signaling cascade [69]. A CaM kinase cascade is important for many normal physiological processes that, when misregulated, can lead to a variety of disease states. These processes include neuronal growth and functions related to brain development, synaptic plasticity, as well as memory formation and maintenance [69, 70]. The expression of TDO2 mRNA in CA2-3,DG showed a tendency towards upregulation at all time points post-ischemia, reaching significance at 48 h (Fig. 4G). However, this transcriptional change was not reflected by a significant change in TDO2 protein levels in either region (Fig. 4H). Tryptophan 2,3-dioxygenase (TDO2) is the rate-limiting enzyme in the kynurenine pathway of tryptophan metabolism [71], which produces metabolites with potent neuromodulatory effects. These include the NMDA receptor agonist quinolinic acid and the broad-spectrum antagonist kynurenic acid (KYNA), which is generally considered neuroprotective [72]. While a post-ischemic increase in TDO2 protein could theoretically lead to increased synthesis of neuroprotective KYNA, our data do not support this mechanism at the protein level. The observed discordance between TDO2 mRNA and protein, and the known complex regulation of KYNA concentration itself [73], suggests this pathway is under tight post-transcriptional control following I/R. Therefore, further studies are required to determine the actual involvement of these proteins in the mechanisms protecting CA2-3,DG neurons.

The group 3 genes (*FZD7*, *ITGB8*, *PHGDH*, and *STC2*) exhibited a distinct response compared to previous groups, showing no significant transcriptional changes in CA2-3,DG after I/R (Fig. 5). This suggests that their regulation in this context may not be directly linked to the Nrf2-driven response seen in Group 1 and 2 genes. However, the observed increases in FZD7, PHGDH, and STC2 protein levels in CA2-3,DG at various time points post-I/R (Figs. 5B, F, H) indicate that post-transcriptional mechanisms may play a role in modulating their abundance and activity. In particular, elevated levels of the FZD7 protein align with other studies implicating activation of Wnt signaling after cerebral ischemia [74]. Given the established role of Wnt signaling in neuroprotection and post-ischemic brain repair, particularly in modulating the adult neural stem cell niche [75], the increased FZD7 protein levels could contribute to the relative resistance of the CA2-3,DG region.

Finally, Group 4 genes (*CXCL12*, *GPC1*, *HRK*, and *LRP8*) displayed inconsistent and region-specific responses to I/R (Fig. 6), defying easy categorization. These genes may be regulated by various factors beyond Nrf2, including interactions with other stress-responsive pathways or region-specific epigenetic modifications. For example, the divergent responses of *CXCL12* in CA1 and CA2-3,DG at 24 h post-reperfusion suggest a dynamic relationship of regulatory mechanisms. CXCL12 has been proven to be broadly neuroprotective, with roles in modulating inflammatory responses [76] and promoting neuronal regeneration after cerebral ischemia [77]. Moreover, CXCL12 has been shown to modulate synaptic transmission to immature neurons during post-ischemic cerebral repair [78]. Changes in *CXCL12* expression early after reperfusion may reflect a mobilization of protective responses, which seem to be less effective in the vulnerable CA1 region. Furthermore, our results demonstrate a delayed increase in GPC1 in CA2-3,DG only at 96 h. Interestingly, GPC1, a heparan sulfate proteoglycan, has been implicated in the clearance of amyloid-β (Aβ) in the brain [79], suggesting a potential neuroprotective role. This delayed upregulation of GPC1 may represent a specific response initiated by the CA2-3,DG region at later stages following I/R, which is not observed in CA1. Similarly, our data indicate a sustained significant decrease in HRK mRNA levels in CA2-3,DG at later time points, contrasted by unchanging levels in CA1 (Fig. 6). Given that HRK, also known as DP5, is a pro-apoptotic protein induced by cellular stress [80], its sustained downregulation in CA2-3,DG could contribute to the region’s resistance to ischemic injury by limiting apoptosis. Finally, *LRP8* mRNA, encoding a receptor involved in Reelin signaling [81, 82], was reduced in CA1 at 72 h and 96 h post-I/R, while in CA2-3,DG it tended to decrease at 96 h, but remained relatively stable (Fig. 6D). The relative preservation of *LRP8* expression in CA2-3,DG, coupled with *HRK* downregulation, could contribute to an environment that favors cell survival in this region following I/R. The functional significance of these varied responses requires further investigation.

### Limitations of the study

This study has several limitations that should be considered when interpreting the findings. Firstly, our initial in silico screening strategy was designed to identify genes with significant basal expression differences between hippocampal subregions. We acknowledge that this approach may have excluded other Nrf2-regulated genes with similar basal expression levels that are nevertheless dynamically regulated following I/R; exploring this latter group represents an interesting avenue for future research. Secondly, this study was conducted exclusively in male gerbils. It is well-established that sex can be a significant biological variable in the response to cerebral ischemia, and future studies will be necessary to determine if the gene expression patterns identified here are conserved in females. Thirdly, while the Mongolian gerbil is an excellent and established model for studying hippocampal ischemia, caution is warranted when extrapolating these findings directly to the complexity of human stroke biology. A key limitation is also the reliance on a mouse transcriptomic database for our initial gene selection, which was then validated in gerbils; species-specific differences in Nrf2 regulation could contribute to some of the discrepancies observed between our in silico predictions and experimental results. Furthermore, due to the limited availability of validated antibodies for Mongolian gerbils, we were unable to assess protein levels for all selected genes. Given the frequent discordance we observed between mRNA and protein expression, the functional implications of changes in transcript levels for which protein data is unavailable should be interpreted with caution. Finally, our study provides a detailed transcriptional and translational profile but lacks direct functional assays (e.g., using siRNA or overexpression models) to causally link the observed expression changes of these specific genes to neuronal survival. Therefore, while the genes identified represent attractive potential drug targets, the path to pharmacological translation is a considerable challenge that will require extensive future investigation.

## Conclusions

Our findings emphasize the complexity of the hippocampal response to I/R injury, revealing distinct patterns of gene expression across different subregions and time points. The observed variability challenges the simplistic view of a uniform neuroprotective mechanism mediated by Nrf2. Instead, it suggests that Nrf2 likely interacts with other signaling pathways in a region-specific and context-specific way. The frequent discordance between mRNA and protein levels further highlights the intricacy of this issue. This illustrates the crucial role of post-transcriptional regulation in shaping the post-ischemic proteome. These results offer new insights into the molecular mechanisms that explain the varying vulnerability of the hippocampal regions to I/R injury. They also suggest that neuroprotection may involve complex interactions between transcriptional and post-transcriptional regulatory networks. Further research into these pathways is critical. Understanding how these identified up- and downregulated genes respond to pharmacological Nrf2 activation holds the potential to unlock novel strategies for enhancing endogenous neuroprotective mechanisms in the hippocampus, ultimately leading to more effective therapies for stroke.

## Supplementary Information

Below is the link to the electronic supplementary material.

Supplementary Material

## Data Availability

Data will be made available on request.

## Abbreviations

ARE: Antioxidant response element
CA2-3,DG: Cornu Ammonis areas 2 and 3, and Dentate Gyrus (of the hippocampus)
Ct: Cycle threshold
ERK: Extracellular signal-regulated kinase
FPKM: Fragments per kilobase per million mapped fragments
GDNF: Glial cell line-derived neurotrophic factor
GCLC: Glutamate-cysteine ligase, catalytic subunit
GCLM: Glutamate-cysteine ligase, modulatory subunit
GPx1: Glutathione peroxidase 1
GSEA: Gene set enrichment analysis
GSH: Glutathione
I/R: Ischemia/Reperfusion
HO-1: Heme oxygenase-1
Keap1: Kelch-like ECH-associated protein 1
KYNA: Kynurenic acid
LDHB: L-lactate dehydrogenase B chain
MAGUK: Membrane-associated guanylate kinase
NAD(P)H: Nicotinamide adenine dinucleotide phosphate
NMDA: N-methyl-D-aspartate
Nrf2: Nuclear factor-erythroid 2-related factor 2
PCs: Pyramidal cells
PMSF: Phenylmethylsulfonyl fluoride
RT-qPCR: Quantitative real-time polymerase chain reaction
RT: Room temperature
scRNA-Seq: single-cell RNA sequencing
SDS-PAGE: Sodium dodecyl sulfate-polyacrylamide gel electrophoresis
TBST: Tris-buffered saline with Tween 20
TIA: Transient ischemic attack
Wnt: Wingless-related integration site

## Declarations

### Generative AI and AI-assisted technologies in the writing process

The writers edited and proofread the document using AI models (Writefull and Google Gemini) to improve readability and language of this work. The authors assumed complete responsibility for the publication’s content and revised and edited it as needed.
