# Supplementary material for: Integrated computational and experimental approach to identify Nrf2-regulated molecular targets in cerebral ischemia
Source: Pharmacol Rep. 2025 Oct 15;77(6):1639–56. doi: 10.1007/s43440-025-00792-9 (PMC12647287; doi:10.1007/s43440-025-00792-9)

**Supplementary Material**

**Supplementary Table 1.** List of Genes and Corresponding Proteins Investigated in the Study.

This table provides a list of the 15 genes analyzed in this study, along with their corresponding protein abbreviations and full protein names. Gene symbols are italicized.

| Gene | Protein Abbreviation | Protein Full Name |
| --- | --- | --- |
| *AIFM2* | FSP1 | Ferroptosis suppressor protein 1 |
| *BRIP1* | BRIP1 | BRCA1 interacting DNA helicase 1 |
| *CAMK1* | CaMK1 | Calcium/calmodulin-dependent protein kinase type 1 |
| *CXCL12* | CXCL12 | C-X-C motif chemokine ligand 12 |
| *FZD7* | FZD7 | Frizzled class receptor 7 |
| *GPC1* | GPC1 | Glypican 1 |
| *HRK* | HRK | Activator of apoptosis harakiri |
| *ITGB8* | ITGB8 | Integrin beta-8 |
| *LRP8* | LRP8 | LDL receptor related protein 8 |
| *MPP3* | MPP3 | MAGUK p55 subfamily member 3 |
| *PHGDH* | 3-PGDH | D-3-phosphoglycerate dehydrogenase |
| *RET* | RET | Proto-oncogene tyrosine-protein kinase receptor Ret |
| *SHISA2* | SHISA2 | Protein shisa-2 homolog |
| *STC2* | STC2 | Stanniocalcin-2 |
| *TDO2* | TDO | Tryptophan 2,3-dioxygenase |

**Supplementary Table 2. Statistical analysis of basal gene and protein expression.**

Detailed statistical results for the one-sample t-tests comparing basal mRNA and protein expression between the CA1 and CA2-3,DG regions of control animals, as presented in Figure 2.

| Gene/Protein | Test | Comparison | t-value (t) | Degrees of Freedom (df) | p-value |
| --- | --- | --- | --- | --- | --- |
| mRNA |  |  |  |  |  |
| *AIFM2* | One-sample t-test | CA1 vs. CA2-3,DG | 5.195 | 6 | 0.020 |
| *BRIP1* | One-sample t-test | CA1 vs. CA2-3,DG | 4.451 | 8 | 0.0021 |
| *CAMK1* | One-sample t-test | CA1 vs. CA2-3,DG | 6.266 | 6 | 0.0008 |
| *CXCL12* | One-sample t-test | (No difference) | n/a | n/a | n/s |
| *FZD7* | One-sample t-test | CA1 vs. CA2-3,DG | 6.375 | 6 | 0.0007 |
| *GPC1* | One-sample t-test | CA1 vs. CA2-3,DG | 4.819 | 8 | 0.0013 |
| *HRK* | One-sample t-test | CA1 vs. CA2-3,DG | 6.534 | 7 | 0.0003 |
| *ITGB8* | One-sample t-test | CA1 vs. CA2-3,DG | 3.517 | 6 | 0.0126 |
| *LRP8* | One-sample t-test | CA1 vs. CA2-3,DG | 3.940 | 7 | 0.0056 |
| *MPP3* | One-sample t-test | CA1 vs. CA2-3,DG | 3.912 | 6 | 0.0079 |
| *PHGDH* | One-sample t-test | CA1 vs. CA2-3,DG | 9.785 | 5 | 0.0002 |
| *RET* | One-sample t-test | CA1 vs. CA2-3,DG | 4.310 | 6 | 0.0050 |
| *SHISA2* | One-sample t-test | CA1 vs. CA2-3,DG | 5.041 | 7 | 0.0015 |
| *STC2* | One-sample t-test | (No difference) | n/a | n/a | n/s |
| *TDO2* | One-sample t-test | CA1 vs. CA2-3,DG | 3.595 | 5 | 0.0156 |
| Protein |  |  |  |  |  |
| 3-PGDH (PHGDH) | One-sample t-test | CA1 vs. CA2-3,DG | 4.749 | 10 | 0.0008 |
| BRIP1 | One-sample t-test | CA1 vs. CA2-3,DG | 2.956 | 8 | 0.0183 |
| CaMK1 | One-sample t-test | CA1 vs. CA2-3,DG | 6.635 | 6 | 0.0006 |
| FSP1 (AIFM2) | One-sample t-test | CA1 vs. CA2-3,DG | 5.309 | 6 | 0.0018 |
| FZD7 | One-sample t-test | CA1 vs. CA2-3,DG | 2.519 | 9 | 0.0328 |
| ITGB8 | One-sample t-test | CA1 vs. CA2-3,DG | 3.222 | 7 | 0.0146 |
| SHISA2 | One-sample t-test | CA1 vs. CA2-3,DG | 5.064 | 7 | 0.0015 |
| STC-2 (STC2) | One-sample t-test | CA1 vs. CA2-3,DG | 2.490 | 6 | 0.0471 |
| TDO (TDO2) | One-sample t-test | (No difference) | n/a | n/a | n/s |

**Supplementary Table 3. Statistical analysis of post-ischemic gene and protein expression.**

Detailed statistical results for the one-way ANOVAs comparing temporal changes in mRNA and protein expression following I/R, as presented in Figures 3-6. Post-hoc analysis was performed using Dunnett's multiple comparison test, comparing each time point to the respective control group.

| Figure in Main Manuscript | Gene / Protein | Region | | Overall ANOVA Result (F(dfb, dfw), p-value) | Significant Time Point vs. Control | p-value summary |
| --- | --- | --- | --- | --- | --- | --- |
| Figure 3 |  | |  |  |  |  |
| 3A | *MPP3* | | CA1 | n/s | None | n/s |
|  | *MPP3* | | CA2-3,DG | F(4, 15) = 5.413,  p = 0.0067 | 72 h  96 h | **  * |
| 3B | *RET* | | CA1 | n/s | None | n/s |
|  | *RET* | | CA2-3,DG | F(4, 12) = 12.61,  p = 0.0003 | 24 h  48 h | ***  ** |
| 3C | *SHISA2* | | CA1 | F(4, 14) = 8.556,  p = 0.0010 | 24 h  48 h | **  * |
|  | *SHISA2* | | CA2-3,DG | F(4, 14) = 15.15,  p < 0.0001 | 24 h  48 h  72 h  96 h | ***  ***  ***  *** |
| 3D | SHISA2 | | CA1 | n/s | None | n/s |
|  | SHISA2 | | CA2-3,DG | n/s | None | n/s |
| Figure 4 |  | |  |  |  |  |
| 4A | *AIFM2* | | CA1 | n/s | None | n/s |
|  | *AIFM2* | | CA2-3,DG | F(4, 14) = 9.041,  p = 0.0008 | 96 h | *** |
| 4B | FSP1 | | CA1 | n/s | None | n/s |
|  | FSP1 | | CA2-3,DG | F(4, 14) = 6.123,  p = 0.0046 | 48 h  72 h  96 h | *  *  *** |
| 4C | *BRIP1* | | CA1 | F(4, 11) = 5.835,  p = 0.0090 | 24 h  48 h  72 h | **  **  * |
|  | *BRIP1* | | CA2-3,DG | F(4, 13) = 12.35,  p = 0.0002 | 48 h  72 h  96 h | ***  **  *** |
| 4D | BRIP1 | | CA1 | F(4, 17) = 4.390,  p = 0.0128 | 72 h | * |
|  | BRIP1 | | CA2-3,DG | F(4, 14) = 8.258,  p = 0.012 | 96 h | *** |
| 4E | *CAMK1* | | CA1 | n/s | None | n/s |
|  | *CAMK1* | | CA2-3,DG |  |  |  |
| 4F | CaMK1 | | CA1 |  |  |  |
|  | CaMK1 | | CA2-3,DG |  |  |  |
| 4G | *TDO2* | | CA1 |  |  |  |
|  | *TDO2* | | CA2-3,DG |  |  |  |
| 4H | TDO | | CA1 |  |  |  |
|  | TDO | | CA2-3,DG |  |  |  |
| Figure 5 |  | |  |  |  |  |
| 5A | *FZD7* | | CA1 |  |  |  |
|  | *FZD7* | | CA2-3,DG |  |  |  |
| 5B | FZD7 | | CA1 |  |  |  |
|  | FZD7 | | CA2-3,DG |  |  |  |
| *...[Continue for all genes/proteins in Fig 5]* |  | |  |  |  |  |
| Figure 6 |  | |  |  |  |  |
| 6A | *CXCL12* | | CA1 |  |  |  |
|  | *CXCL12* | | CA2-3,DG |  |  |  |
| *...[Continue for all genes in Fig 6]* |  | |  |  |  |  |

**Supplementary Figure 1.**

The figure illustrates the division of the mouse hippocampus into dorsal and ventral regions and the specific cell types analyzed, as adapted from the Hipposeq RNA-seq database (http://hipposeq.janelia.org/, accessed 12.01.2025) (Cembrowski et al., 2016). The regions include: dorsal CA1, CA2, and CA3 pyramidal cells (PCs); ventral CA1 and CA3 PCs; dorsal and ventral dentate gyrus (DG) granule cells (GCs); and dorsal DG mossy cells (MCs)


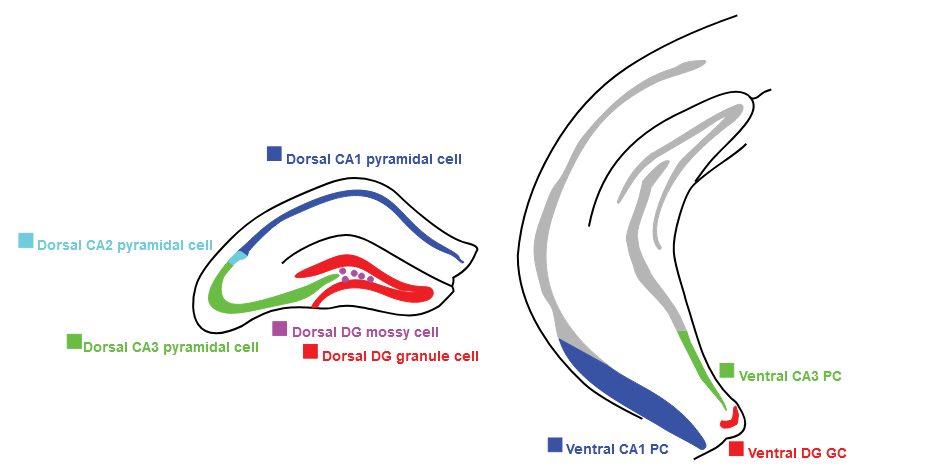


**Supplementary Figure 2.**

Heatmap of the logarithmic fold change in expression of Nrf2-regulated genes between the dorsal CA1 region and ventral CA1, dorsal CA2, dorsal CA3, ventral CA3, dorsal DG, ventral DG, and MC in the mouse hippocampus. Color indicates the log10 of the fold change in expression. Light green and dark green represent a 10x and 100x increase in expression in a given region relative to the dorsal CA1 region, respectively. Red indicates a 100x decrease in expression. Gray indicates a lack of gene expression in a given region.


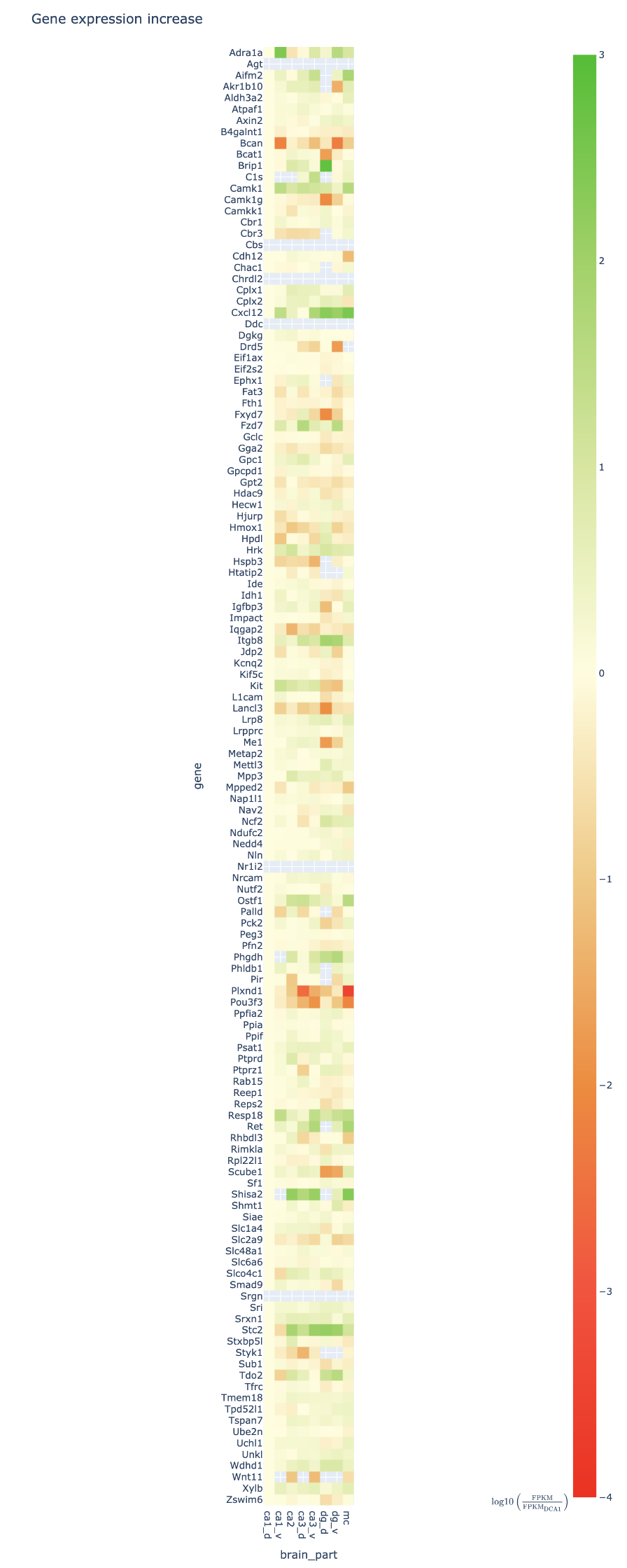

Supplement: Supplementary file 1 — Supplementary Material [file 43440_2025_792_MOESM1_ESM.docx]
